# e-Cigarette Use and the Cessation of Tobacco Cigarette Smoking: Protocol for an Umbrella Review

**DOI:** 10.2196/47711

**Published:** 2023-08-10

**Authors:** Renee O'Leary, Riccardo Polosa

**Affiliations:** 1 Center of Excellence for the Acceleration of Harm Reduction University of Catania Catania Italy

**Keywords:** e-cigarettes, electronic nicotine delivery systems, ENDS, umbrella review, cessation, systematic reviews, A Measurement Tool to Assess Systematic Reviews, version 2, AMSTAR2, review methodology, systematic, cigarette, smoking, smoker, quit, meta-analysis, meta-analyses

## Abstract

**Background:**

Electronic nicotine delivery systems (ENDS), commonly called e-cigarettes, have been examined in clinical studies for their effects on tobacco smoking cessation. In the past 2 years, a dozen or more systematic reviews on ENDS and cigarette smoking cessation have been published that present differing conclusions and recommendations on the use of ENDS.

**Objective:**

Our umbrella review aims to synthesize the findings from current systematic reviews to investigate the quit rates and the percentage of participants abstinent from cigarette smoking using ENDS. Additionally, we will examine the quit rates with ENDS in comparison to other established cessation treatments.

**Methods:**

The search will retrieve systematic reviews that include both clinical trials and experimental studies on the use of ENDS for smoking cessation. We will also include nonrandomized cohort studies that track ENDS use and the subsequent abstinence from smoking. Databases searches will be conducted in Embase, Scopus, PubMed, and 7 additional registries. Secondary searches will include reference checking, citation chasing, and consultations with topic experts. Two reviewers will perform a title and abstract exclusion followed by a full-paper inclusion process. Data extraction will be conducted by 1 reviewer and completely checked by a second reviewer. Each systematic review will be assessed by 2 reviewers for methodological quality using AMSTAR2 (A Measurement Tool to Assess Systematic Reviews, version 2) and for reporting bias using categories from the Centre for Evidence-Based Medicine’s Catalogue of Bias. Unreported discrepancies between the protocol and the published review will be identified.

**Results:**

The umbrella review started on March 1, 2023. At the time of publication, the study selection was being conducted and the pilot testing of the data extraction and bias assessment forms were in progress. The review is expected to be completed by December 31, 2023, followed by the submission of the review for journal publication. A second-order meta-analysis will calculate the range and average of quit rates for ENDS. A vote counting of the direction of effect, based on quit rates, will be used to present the relative effectiveness of ENDS for smoking cessation compared to other cessation treatments (including no treatment). A citation matrix will list primary studies with their bias ratings from all the systematic reviews. The effect of overlapping studies between the systematic reviews will be calculated using the corrected coverage area analysis. A sensitivity analysis will examine the impact of the intensity of cessation treatment on quit rates. Depending on the availability of data, subgroup analyses will be conducted based on participants’ gender, age, prior quit attempts, and nicotine dependence. The strength or weakness of the evidence synthesis will be assessed using a stratification of evidence technique. Reporting bias will be presented with a tabulation of bias indicators. Publication bias will be assessed.

**Conclusions:**

The use of ENDS for smoking cessation is a highly controversial subject. Through an exhaustive synthesis of the available data, we will present the quit rates of cigarette smoking cessation obtained with ENDS and how they compare to quit rates obtained from other established cessation treatments. The critical quality and bias assessment of the systematic reviews will indicate the most reliable sources to inform treatment considerations and policy development.

**Trial Registration:**

PROSPERO CRD42023406165; https://tinyurl.com/4ekzpbrj

**International Registered Report Identifier (IRRID):**

PRR1-10.2196/47711

## Introduction

e-Cigarettes, termed electronic nicotine delivery systems (ENDS), were invented in 2003 to be a cigarette cessation device [1]. The substantially reduced toxicant profile of ENDS compared to cigarettes [2,3] has motivated researchers to investigate its potential for supporting cigarette smoking cessation. As the number of clinical studies piled up, the first systematic review on ENDS for smoking cessation was published in 2014 by the Cochrane Group [4], and 21 systematic reviews were published by 2017 [5]. Even after excluding studies with a cessation outcome of less than 6 months’ duration, the number of primary studies included in 2 current systematic reviews was 61 and 64, respectively [6,7]. Our preliminary search in PubMed for systematic reviews published since 2021 retrieved 20 potential publications.

Another systematic review is unlikely to make a significant contribution until more experimental studies are published. Our purpose for conducting an umbrella review is to provide the widest overview of the clinical evidence on the use of ENDS and their impact on cigarette smoking abstinence for an audience of researchers and clinicians highly polarized over the use of ENDS [8-10]. An umbrella review systematically searches and retrieves systematic reviews, critically appraises them, and synthesizes their results [11].

We have 4 research questions: 2 focus on cigarette smoking abstinence outcomes, and the other 2 focus on the quality of the evidence. These questions are as follows:

1. Are ENDS more, less, or equally effective in quit rates (ie, the percentage of participants who abstained from smoking), compared to other cigarette smoking cessation treatments?

2. What is the range of quit rates with ENDS in absolute numbers?

3. How weak or strong is the evidence base on the effectiveness of ENDS for smoking cessation?

4. Is reporting bias present in systematic reviews of ENDS for smoking cessation?

## Methods

### Structure of Review

Our protocol has been designed with reference to the Methods for Overviews of Reviews (MOoR) framework [11,12]. It has 20 steps with 54 substeps. Our protocol is reported based on the checklist provided in the recently published Preferred Reporting Items for Overviews of Reviews (PRIOR) statement by Gates et al [13]. It consists of 27 main items, 46 in total, and is specifically designed for health care interventions.

The population, intervention, comparator, and outcome (PICO) criteria below define the scope of our umbrella review for evidence on cigarette smoking cessation or abstinence with the use of ENDS. These criteria are as follows:

- Population: adults who formerly smoked and currently use ENDS or clinical trial participants who smoked cigarettes at baseline before using ENDS.
- Intervention: personal use of ENDS or ENDS use in a clinical trial.
- Comparator: cigarette smoking cessation treatments, including no treatment.
- Outcome: (1) quit rates, that is, the percentage of participants who abstained from smoking or quit smoking and (2) the quit rate with ENDS compared to other cessation treatments.

### Search and Selection Processes

Database search sources will include Embase (Ovid), Scopus (Elsevier), PubMed, Cochrane Database of Systematic Reviews, PROSPERO, and Epistemonikos. For grey literature, database search sources will include MedNar, National Technical Information Service, WorldWideScience, and Open Grey.

The search parameters include keywords related to the intervention (eg, “cessation” OR “quit” OR “abstinence”), ENDS (eg, “e-cigarettes” OR “electronic nicotine” OR “electronic cigarettes” OR “vaping” OR “vape”), and the study design (eg, “systematic review”). The search fields include title, abstract, and keywords. A filter for systematic reviews will be applied, whenever available. One researcher will review the applicable keywords, fields, and filters specific to the database. Syntaxes will vary by database. The search syntaxes for each database will be recorded with a screenshot, counted, and dated; the retrieved records will be downloaded into an EndNote library. The library will be deduplicated, and the count will be recorded. The publication date starts from 2021 for 2 reasons. First, current systematic reviews are most likely to include the most recently published primary studies. Second, a preliminary scoping search was conducted to explore the possibility of an umbrella review study, and it retrieved 20 potentially eligible systematic reviews published from 2021, which is a substantial number for inclusion in an umbrella review.

Two researchers will independently screen the records by title and abstract (or summary). They will include the systematic reviews that cover the PICO components. Discrepancies will be resolved through discussion and referred to the project leader for a final disposition, if necessary. The interrater agreement and the count of excluded studies will be reported.

The next stage of the selection process is a full-paper review. Two reviewers will independently examine each study for inclusion and exclusion based on the criteria presented in Table 1.

Industry-funded studies, if any, will be discussed separately from the analyses.

Two researchers will independently evaluate the reviews for inclusion. Discrepancies will be resolved by discussion, and if unresolved, will be referred to the project leader for a final disposition and recorded for interrater reliability. All systematic reviews excluded during the full-paper examination will be listed, along with the reason for exclusion, in an appendix.

In a second round of searching, the systematic reviews selected for inclusion will be citation chased (snowball search) in Google Scholar by 2 researchers who independently examine the title and abstract of the papers. The selected papers will be downloaded and organized as a separate group within the library. Discrepancies between the 2 retrievals will be resolved through discussion or referred to the project leader for a final disposition, and the interrater agreement will be reported. Systematic reviews retrieved from this search will be selected using the criteria for full-paper review. The completed full-paper search will be reviewed by 2 topic experts, who will provide recommendations for any additional publications.

**Table 1:** Inclusion and exclusion criteria.

Criteria	Inclusion	Exclusion
Article type	Systematic review	All other study designs
Language	English, French, Italian, and other languages that may be translated	Reviews without an English abstract or summary
Publisher	Academic journals, government reports, and medical organizations	Predatory journals (not indexed in PubMed or Directory of Open Access Journals)
Primary study designs	Clinical trials, experimental studies, observational cohort studies, and randomized or nonrandomized studies	Cross-sectional studies, surveys, case studies, and qualitative studies
Population	Adults who use ENDS^a^, who smoke, or who have stopped smoking cigarettes	Youth or participants who did not smoke before using ENDS
Data	Quit rates with ENDS and comparison of quit rates with ENDS and with other treatments	Reduction of cigarette use and dual use of ENDS and cigarettes
Duration of cessation	Explicitly defined	Duration not specified
Bias assessment of primary studies	Individual primary studies assessed using any method	No bias assessment of individual primary studies
Analysis or synthesis method	Meta-analysis and tabulation of quit rates across primary studies	Solely narrative or descriptive

^a^ENDS: electronic nicotine delivery systems.

### Data Collection

The data extraction and quality assessments of a systematic review and its supplementary materials will be conducted simultaneously.

Data extraction will be performed using a prespecified data extraction form covering bibliographic information; funders; locality and setting; databases searched and dates; inclusion and exclusion criteria; outcome definition; primary studies with their bias assessments; data analyses (including sensitivity analyses); meta-analyses; any subgroup analyses; and the conclusion, quoted directly from the study. The specific items for data extraction will be identified by the research team. The form will be pilot-tested by the research team on 3 reviews and revised as needed. Data collection will be conducted by 1 researcher, and a second reviewer will cross-check the data extractions.

In cases where data items are missing, we will contact the author. Reviews lacking multiple data items may be excluded at this stage of the umbrella review. Any studies excluded for missing data items will be reported.

### Quality Assessment

After completing the data extraction, the 2 reviewers will independently complete the AMSTAR2 (A Measurement Tool to Assess Systematic Reviews, version 2) checklist [14] to assess the overall confidence in the results of the review as high, moderate, low, or critically low. The research team will decide in advance which of the 16 domains will be designated as noncritical weaknesses or critical weaknesses. The rating assigned to these domains will be used as a proxy for assessing the risk of bias, with higher ratings indicating a lower risk of bias. Discrepancies in scoring will be discussed and referred to the project leader for disposition, if necessary. The interrater reliability will also be reported.

After completing the data extraction and the AMSTAR2 checklist, 2 reviewers will independently assess each review for reporting bias. Reporting bias will be identified by the following indicators: spin bias of nonsignificant findings, omitted findings, one-sided reference bias, framing by over- or underemphasis of outcomes, overreliance on *P* values, and data discrepancies [15]. The reporting of the review will be compared to its protocol or trial registration, and any deviations will be noted. Any indicators of reporting bias will be verified by the project leader.

Before conducting the syntheses, we will conduct a sensitivity analysis to examine the overlap of primary studies across the systematic reviews. Analyzing overlaps is critical because they can result in certain studies being overweighted in the analysis. Currently, there is no one standard method in an umbrella review to correct or weight the meta-analysis for the overlap of primary studies across reviews [16]. Based on its long-term use and validation, we have selected the corrected covered area (CCA) [17]. The formula is CCA=(*N*-*r*)/(*rc*-*r*) where *N* is the total number of publications, *r* is the number of index studies, and *c* is the number of reviews. To calculate the CCA, a citation matrix of primary studies will be tabulated, and it will be included in our published review. The CCA score is classified as slight (0%-5%), moderate (6%-10%), high (11%-15%), and very high (>15%). The CCA analysis will be conducted with the 5-step process developed by Hennessy and Johnson [18].

The CCA score will inform the selection of systematic reviews for the meta-analyses using the decision tool developed by Pollock et al [19]. We will include the highest rated review by AMSTAR2 score where the reviews have a higher level of overlap. If a 90% overlap in primary studies is found among 3 or more reviews, they will be included together in a subgroup analysis.

Before conducting the syntheses, the primary study data items will be tracked across the reviews to identify any data discrepancies. Data discrepancies will be corrected by examining the primary study. This process constitutes data cleaning.

## Results

### Overview

The umbrella review started on March 1, 2023. At the time of publication, the study selection was being conducted and the pilot testing of the data extraction and bias assessment forms were in progress. The review is expected to be completed by December 31, 2023, followed by the submission of the review for journal publication.

The search and selection results will be reported with the PRIOR diagram.

A table of review characteristics will display pertinent information, data, and the AMSTAR2 ratings. Comprehensive review tables and the AMSTAR2 scoring will be presented in supplementary materials.

A citation matrix will present all the primary studies included in the systematic reviews and will list the bias rating given to the primary study by each review.

Analyses of heterogeneity across systematic reviews will be conducted on inclusion and exclusion criteria and the comparator used (eg, other cigarette smoking cessation treatments or no quit intention).

A check for publication bias based on quit rates will be conducted if there are 10 or more reviews with meta-analysis data available. A funnel plot will be graphed and analyzed.

Depending on the availability of data in the systematic reviews, we will conduct sensitivity analyses on 3 effect modifications related to smoking cessation: nicotine dependency, prior quit attempts, and treatment intensity (eg, counseling vs no support). A total of 3 data points will be required to conduct the analysis. If an effect modification is reported in fewer than 3 reviews, the findings will be presented in a narrative summary.

### Planned Syntheses—Quit Rates and Comparative Quit Rates of Cessation Treatments

For the first research question, which pertains to the relative effectiveness of ENDS compared to other methods (including no method), we will conduct a vote counting of direction of effect for each individual cessation treatment.

For the second research question, which pertains to the quit rate for ENDS, we will conduct a second order meta-analysis by combining the odds ratios for quit rates obtained from various systematic reviews. The data from the meta-analyses in the reviews will be converted to odds ratios by using the conversion formulas listed by Fusar-Poli and Radua [20] in cases where they were not computed as such.

Each of the syntheses conducted for the 2 cessation questions will be tested with 3 sensitivity analyses: length of cessation (any vs >6 months vs longer durations), the AMSTAR2 rating (high rating vs all others), and the inclusion or exclusion of industry-funded primary studies in the systematic review.

For each of the cessation analyses, a subgroup analysis by gender and age will be conducted, where feasible.

### Planned Syntheses—Quality of Evidence

For the third research question, which focuses on the strengths or weaknesses of the evidence, we will evaluate the strength of the evidence base by conducting a stratification of evidence with the criteria published by Fusar-Poli and Radua [20]. This method rates a meta-analysis as convincing, highly suggestive, suggestive, weak, or nonsignificant, as stratified by the number of cases, the *P* values, the *I*^2^ values, and biases. The method will require a slight adaptation to suit the requirements of an umbrella review.

For question 4, which is related to reporting bias, we will tabulate the number of incidences identified for each reporting bias indicator (as described above), grouped by review and for each bias indicator.

### Additional Items

To assess the overall confidence in the findings of the umbrella review, a scoring system will be used based on the number of study designs included and their AMSTAR2 rating, with a modification of the GRADE (grading of recommendations, assessment, development, and evaluations) system.

The “Discussion” section will include a summary of the main findings, any contradictory findings between the systematic reviews, limitations of the systematic reviews, limitations of the umbrella review, recommendations for future research, and the relevance of the findings to clinicians and policy makers.

All deviations from this protocol will be documented and justified in the published study.

## Discussion

Regarding the projected quit rates for smoking cessation, we expect them to be low, which unfortunately is the case for all cigarette smoking cessation treatments.

Our umbrella review will be subject to limitations. First, we will have a small research team of 4 members. Fortunately, the team has expertise in systematic and umbrella reviews with decades of research experience on ENDS. Regarding staffing constraints, we are well aware from prior experience that the identification of reporting bias is time intensive, but in our estimation, it is absolutely essential for credibility in our highly contested field.

Our dissemination plans are also subject to funding limitations. As a minimum, we will publish our umbrella review in an open-access journal and produce a white paper in plain language with infographics and post it on our research unit’s website. We will also submit it for conference presentations after publication if team members are available. Low-cost avenues of knowledge translation will be considered as time permits.

The methods and processes specified in this protocol are all aimed at achieving one goal: to identify data on the effectiveness of ENDS for cigarette smoking cessation. Our purpose is to provide clinicians, policy makers, and individuals who smoke with the published evidence, enabling them to make evidence-informed decisions. With 15 or more systematic reviews published in just the past 2 years on ENDS for cigarette smoking cessation, the literature is ripe for an umbrella review.

## Abbreviations

AMSTAR2: A Measurement Tool to Assess Systematic Reviews, version 2
CCA: corrected coverage area
ENDS: electronic nicotine delivery systems
GRADE: grading of recommendations, assessment, development, and evaluations
MOoR: Methods for Overviews of Reviews
PICO: population, intervention, comparator, outcome
PRIOR: Preferred Reporting Items for Overviews of Reviews

## Data Availability

All data will be available in the systematic reviews. For Open Science, the completed data extraction forms, the AMSTAR2 checklists, and bias reports will be uploaded to Zenodo.
